# A Practical Treatise on Fractures and Dislocations

**Published:** 1884-12

**Authors:** 


A PRACTICAL TREATISE ON FRACTURES AND DISLOCATIONS. *By* FRANK HASTINGS HAMILTON, A.B., A.M., M.D., LL.D. *Late Professor of Surgery in Bellevue Hospital Medical College, and Surgeon to Bellevue Hospital, New York. Seventh American Edition, Revised and Improved. Octavo, pp. xxxi, 1005. Philadelphia:* HENRY C. LEA'S SON & CO. 1884.

Professor Hamilton's book is so widely and favorably known that the reviewer's task is easy. Observations drawn from more recent personal experience and a résumé of more lately recorded facts have resulted in a considerable enlargement of the size of the volume. As thus improved, we have no hesitation in pronouncing the book one of the best—if not the best—of its kind in the English language.

